# Advanced parental age is an independent risk factor for term low birth weight and macrosomia

**DOI:** 10.1097/MD.0000000000029846

**Published:** 2022-06-30

**Authors:** Yoo Hyun Chung, In Sun Hwang, Gyul Jung, Hyun Sun Ko

**Affiliations:** a Department of Obstetrics and Gynecology, Daejeon St. Mary’s Hospital, College of Medicine, the Catholic University of Korea, Suwon, Republic of Korea; b Department of Obstetrics and Gynecology, College of Medicine, the Catholic University of Korea, Seoul, Republic of Korea; c Department of Obstetrics and Gynecology, Seoul St. Mary’s Hospital, College of Medicine, The Catholic University of Korea, Seoul, Republic of Korea.

**Keywords:** birth weight, low birth weight, macrosomia, maternal age, paternal age, term birth

## Abstract

We aimed to investigate association between parental age and the risks of term low birth weight and macrosomia.

This was a retrospective cohort study using a national database including 2,245,785 term singleton live births with complete parental age data. Old parental age was defined as 35 years or older. Odd ratios (OR) for term low birth weight and macrosomia were analyzed using univariate and multivariate logistic regression analysis.

Neonatal sex, maternal occupation, parity, nationality, age, and paternal age were significant factors of term low birth weight and macrosomia, in univariate analysis. In multivariate analysis, old maternal age (≥35 years old) showed increased odds of term low birth weight and macrosomia (aOR = 1.122, 95% CI: 1.083 –1.162; and aOR = 1.166, 95% CI: 1.143 – 1.189, respectively). Similarly, old paternal age (≥35 years old) showed increased odds of term low birth weight and macrosomia (aOR = 1.090, 95% CI: 1.058 –1.122; and aOR = 1.101, 95% CI: 1.083 – 1.119, respectively). Maternal education that lasted more than 12 years had reduced odds of term low birth weight and macrosomia (OR = 0.817, 95% CI: 0.792 –0.842; and OR = 0.894, 95% CI: 0.879 – 0.91, respectively). Paternal education that lasted more than 12 years also had reduced odds of term low birth weight and macrosomia (OR = 0.865, 95% CI: 0.84 –0.892; and OR = 0.897, 95% CI: 0.881 – 0.913, respectively).

This study suggests that not only maternal age but also paternal age are significantly associated with term low birth weight and macrosomia. In addition, parental education levels are also associated with term low birth weight and macrosomia.

## 1. Introduction

Advanced maternal age is defined as a pregnancy for women that are over 35 years.^[1]^ According to data from the Korea National Statistical Office in 2019, the number of mothers aged 35 years or older reached 33.9%, which increased by 1.6% from the previous year.^[2]^ Several studies have reported that advanced maternal age is a risk factor for many complications in pregnancy, including low birth weight (LBW) or macrosomia of neonates.^[3–7]^However, only a limited number of studies have reported on the relationship between pregnancy complications and paternal age. Advanced paternal age has been associated with genetic mutations, miscarriage, preeclampsia and some birth defects.^[8]^

LBW is defined as <2500 g.^[9]^ Birth weight has a direct relationship with mortality and morbidity as well as inhibited growth, cognitive development, and chronic diseases.^[10,11]^Close observation of intrauterine growth is essential because LBW infants are at an increased risk of developmental disorders due to their smaller size compared with their peers, and of having chronic diseases later in life.^[11]^ According to statics from the Korean National Statistical Office in 2019, LBW accounts for 6.6% of all birth, which increased by 0.4% compared to the previous year.^[2]^

Macrosomia is defined as a birth weight greater than 4000 g or 4500 g.^[12]^ Macrosomia can lead to complications such as dystocia, controlled labor, cesarean delivery, postpartum hemorrhage, and vaginal laceration.^[11]^ Although it has been suggested that advanced maternal age is a risk factor for macrosomia, there are no data regarding the association between advanced paternal age and macrosomia.^[13]^

This study evaluated whether maternal and paternal age are independent risk factors for term low birth weight (TLBW) and macrosomia at a population level in Korea.

## 2. Materials and methods

This was a retrospective cohort study that used deidentified Korean Vital Statistics Birth Certificate data from 2010 to 2015.^[13]^ Korean Vital Statistics is a nationwide database that was developed to understand birth, death, marriage, and divorce.

Raw data on all newborns delivered from 2010 to 2015 (470,171 in 2010, 471,265 in 2011, 484,550 in 2012, 436,455 in 2013, 435,435 in 2014, and 438,420 in 2015) were analyzed. Our results were based on 2,245,785 births, after exclusions. Pregnancy dating was determined using the best obstetric estimate as opposed to the last menstrual period. Gestational age (GA) was referred to by interval, using completed weeks. For example, a GA of 40 weeks indicates 40 weeks plus 0 to 6 days. Birth weight was measured to the nearest 10 g. This study used anonymous registry data and obtained approval from the Institutional Review Board of the Catholic University of Korea (KC17ZESI01).

### 2.1. Inclusion and exclusion criteria

The study population included all singleton deliveries to mothers between 38 0/7 and 41 6/7 weeks of gestation. Newborns with an unknown body weight or GA (n = 6846), unknown maternal or paternal age (n = 17,380), GA < 38 weeks or > 41 weeks (n = 429,321), multiple pregnancies (n = 91,370), unknown maternal education duration (n = 5412), unknown paternal education duration (n = 4434), unknown maternal employment status (n = 13,816), extreme maternal age (<20 or >45 years old, n = 1572), and extreme paternal age (<0 or >50 years old, n = 14,440) were excluded. Some exclusions (n = 88,548) were duplicated.

### 2.2. Outcome measures

Among the newborns that were born between 38 and 41 gestational weeks, those with a birth weight under 2500 g were defined as TLBW, those with a birth weight between 2500 g and 4000 g were defined as normal body weight (NBW), and those with a birth weight of 4000 g or above were defined as having macrosomia. Term deliveries at 37 weeks of gestation were excluded from this analysis because deliveries at 37 weeks are usually indicated by maternal or fetal complications, and birth statistics do not include data regarding complications of the mother and neonate. Old maternal or paternal ages were defined as 35 years old or older. Because it takes 12 years for graduation of high school in South Korea, high education level was defined as education over 12 years. Univariate analysis and multivariate analysis were performed to examine the effects of maternal and paternal ages on the risk of TLBW and macrosomia.

### 2.3. Statistical analyses

Statistical calculations were performed using SPSS version 24.0 (SPSS, Inc., Chicago, IL). Categorical data are presented as the number (%) and compared using the χ^2^ test. Continuous variables are presented as the mean ± standard deviation and analyzed using independent *t*-test. In an effort to assess the independent predictors of TLBW and macrosomia, we calculated the odds ratios (ORs) and 95% confidence intervals (CIs) using logistic regression models adjusted for maternal age, paternal age, gestational age, parity, neonatal sex, maternal employment status, and parents’ education level. Maternal ages and gestational ages are presented to one decimal place. Statistical significance was considered as *P* < 0.05 or a 95% CI of the OR that did not include 1.

## 3. Results

**Figure 1.**
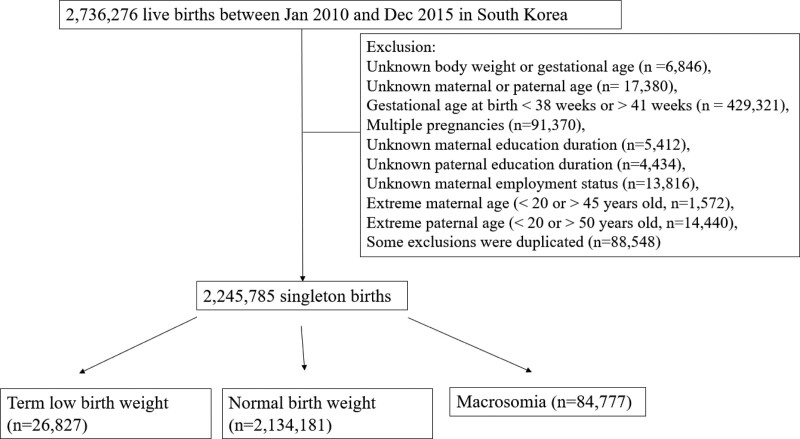
A flow chart illustrating the participant selection process, using the Korean National Health Insurance Service-National Sample Cohort. Out of a total of 2736,276 participants, 2,245,785 participants were collected.

Our analysis included 2,245,785 term singleton live births aged 38 to 41 weeks’ gestation in Korea between 2010 and 2015, after applying the exclusion criteria (Fig. 1).

### 3.1. Clinical characteristics

Among the 2,245,785 singleton births, 26,827 (1.2%) neonates were included in the TLBW group, and 84,777 (3.8%) were included in the macrosomia group. There were significant differences in gestational age at birth, parity, percentage of old maternal age (≥35 years) and old paternal age (≥35 years), maternal nationality, maternal occupation and education levels between NBW and TLBW groups, and between NBW and macrosomia groups (*P* < 0.001, all) (Table 1). The percentages of female neonates, nulliparity, old maternal age (≥35), and non-Korean maternal nationality were highest in the TLBW group, and the percentages of maternal occupation, maternal and paternal education that lasted for more than 12 years were highest in the NBW group. The percentage of old paternal age (≥35 years) was highest in the macrosomia group.

**Table 1:** Baseline characteristics.

	Normal BW (n = 2134,181)	TLBW (n = 26,827)	Macrosomia (n = 84,777)	*P*-value*	*P*-value†
Gestational age (weeks)	39.12 ± 0.92	38.60 ± 0.76	39.45 ± 0.92	<0.001	<0.001
Birth weight (kg)	3.28 ± 0.32	2.33 ± 0.15	4.17 ± 0.18	<0.001	<0.001
Maternal age (years)	31.14 ± 3.99	31.17 ± 4.36	31.61 ± 3.95	0.219	<0.001
≥35 years old	395,815 (18.5)	5510 (20.5)	18,715 (4.5)	<0.001	<0.001
Paternal age (years)	33.74 ± 4.39	33.9 ± 4.69	34.16 ± 4.42	<0.001	<0.001
≥35 years old	849,660 (39.8)	11,138 (41.5)	37,337 (44.0)	<0.001	<0.001
Nulliparity (%)	1134,393 (53.2)	17,041 (63.5)	41,683 (49.2)	<0.001	<0.001
Female neonate (%)	1059,008 (49.6)	16,738 (62.4)	29,504 (34.8)	<0.001	<0.001
Maternal occupation (%)	752,986 (35.3)	8751 (32.6)	29,080 (34.3)	<0.001	<0.001
Maternal education > 12 years (%)	1564,571 (73.3)	17,982 (67.0)	59,637 (70.3)	<0.001	<0.001
Paternal education > 12 years (%)	1568,615 (73.5)	18,174 (67.7)	59,885 (70.6)	<0.001	<0.001
Non-Korean Maternal nationality	84,632 (3.9)	1402 (5.2)	3115 (3.7)	<0.001	<0.001

All values are means ± SD, or n (%). *P*-values were calculated by *t*-tests or chi square tests.

BW = birth weight; TLBW = term low birth weight.

* Statistical comparison between Normal BW and TLBW.

† Statistical comparison between Normal BW and Macrosomia.

### 3.2. Univariate analysis

In the univariate analysis, the female neonates showed a significantly higher odd of TLBW (OR = 1.684, 95% CI: 1.643–1.727) (Table 2). Foreign nationality of mothers, old maternal age, and old paternal age also presented increased odds of TLBW (OR = 1.369, 95% CI: 1.297–1.445, OR = 1.135, 95% CI: 1.102–1.170 and OR = 11.073, 95% CI: 1.047–1.110, respectively). Maternal and paternal education that lasted more than 12 years, maternal occupation, and a previous history of delivery presented significantly lower odds of TLBW (OR = 0.740, 95% CI: 0.721–0.759; OR = 0.757, 95% CI: 0.738–0.777; OR = 0.888, 95% CI: 0.866–0.911; and OR = 0.652, 95% CI: 0.635–0.668, respectively).

**Table 2:** Univariate analysis for the risk of term low birth weight or macrosomia.

	Term low birth weight				Term macrosomia			
			95% CI for odds ratio				95% CI for odds ratio	
Characteristics	*P*-value	Odds ratio	Upper	Lower	*P*-value	Odds ratio	Upper	Lower
Female neonate	1.684	1.643	1.727	<0.001	0.542	0.534	0.55	<0.001
Maternal occupation	0.888	0.866	0.911	<0.001	0.958	0.944	0.972	<0.001
Multiparity	0.652	0.635	0.668	<0.001	1.173	1.157	1.189	<0.001
Foreign maternal nationality	1.369	1.297	1.445	<0.001	0.947	0.913	0.982	0.003
Maternal education > 12 years	0.74	0.721	0.759	<0.001	0.867	0.854	0.881	<0.001
Paternal education > 12 years	0.757	0.738	0.777	<0.001	0.864	0.851	0.877	<0.001
Maternal age ≥ 35 years old	1.135	1.102	1.17	<0.001	1.244	1.244	1.265	<0.001
Paternal age ≥ 35 years old	1.073	1.047	1.11	<0.001	1.19	1.173	1.206	<0.001

CI = confidence interval.

Male neonate, no maternal occupation, nulliparity, mother’s nationality (Korean), education less than 12 years, young maternal age (<35 years old), and young paternal age (<35 years old) were used as references.

However, old maternal age, paternal age and multiparity also showed significantly higher odds of macrosomia; the odd ratios of macrosomia were significantly increased with a maternal age ≥ 35 years, a paternal age ≥ 35 years, and multiparity (OR = 1.244, 95% CI: 1.224–1.265; OR = 1.190, 95% CI: 1.173–1.206; and OR = 1.173, 95% CI: 1.157–1.189, respectively). Maternal and paternal education that lasted more than 12 years, maternal occupation, and female neonates showed significantly lower odd ratios of macrosomia (OR = 0.867, 95% CI: 0.854–0.881; OR = 0.864, 95% CI: 0.851–0.877; OR = 0.958, 95% CI: 0.944–0.972; and OR = 0.542, 95% CI: 0.534–0.550, respectively).

### 3.3. Multivariate analysis

In the multivariate logistic regression analysis, after adjusting for neonatal sex, gestational weeks at delivery, maternal occupation, and parity, old maternal age had significantly increased odds of TLBW and macrosomia, (aOR = 1.122, 95% CI: 1.083–1.162; and aOR = 1.166, 95% CI: 1.143–1.189, respectively). In multivariate analysis, old paternal age also had significantly increased odds of TLBW and macrosomia (aOR = 1.090, 95% CI: 1.058–1.122; and aOR = 1.101, 95% CI: 1.083–1.119, respectively) (Table 3). Maternal education that lasted more than 12 years showed reduced odds of TLBW and Term macrosomia. (aOR = 0.817, 95% CI: 0.792–0.842; and aOR = 0.894, 95% CI: 0.879–0.91, respectively). Paternal education that lasted more than 12 years also showed reduced odds of TLBW and macrosomia (aOR = 0.865, 95% CI: 0.84–0.892; and aOR = 0.897, 95% CI: 0.881–0.913, respectively).

**Table 3:** Multivariate logistic regression analysis for the risk of term low birth weight and macrosomia.

	Term low birth weight				Term macrosomia			
			95% CI for odds ratio				95% CI for odds ratio	
Characteristics	*P*-value	odds ratio	upper	lower	*P*-value	odds ratio	upper	lower
Maternal age ≥ 35 years old	<0.001	1.122	1.083	1.162	<0.001	1.166	1.143	1.189
Paternal age ≥ 35 years old	<0.001	1.090	1.058	1.122	<0.001	1.101	1.083	1.119
Maternal education > 12 years	<0.001	0.817	0.792	0.842	<0.001	0.894	0.879	0.91
Paternal education > 12 years	<0.001	0.865	0.84	0.892	<0.001	0.897	0.881	0.913

CI = confidence interval.

Adjusted for neonatal sex, gestational weeks at delivery, maternal occupation, and parity. Education < 12 years, young maternal age (<35 years), and young paternal age (<35 years) were used as references.

## 4. Discussion

This study demonstrated that both maternal and paternal age with ≥35 years were associated with TLBW and term macrosomia.

Previous studies have reported that prenatal complications are related to advanced paternal age, which included fetal loss and congenital malformations.^[14,15]^ Although several studies have suggested that paternal factors have an effect on perinatal outcomes,^[8,15,16]^ only a few studies have investigated the effects of paternal age on LBW and macrosomia in Asia. In this study, we analyzed a large dataset to determine whether paternal age is a risk factor for LBW and macrosomia.

Parental age has been increased globally, which cannot be reversed. Although it is unknown that LBW or macrosomia can be prevented through systematic prenatal care, such as balanced nutrition, exercise, and appropriate maternal weight gain, the association between advanced parental age and neonatal birth weight need to be clarified.^[16]^ There is currently no uniform definition of advanced paternal age, although a male that is 40 years of age or older at the time of conception is most frequently used to define advanced paternal age.^[14]^ There is some evidence that advanced paternal age is associated with increased genetic and epigenetic risk to offspring.^[14]^ However, the precise paternal age at which risk develops and the range of the risk are poorly understood, especially in Asians. Reports have indicated that an increase in paternal age can lead to errors in DNA replication during the spermatogenesis process, which can cause problems in implantation and subsequent growth.^[15,17]^Other previous studies also suggested that advanced paternal age increases concern for sperm quality, assisted reproductive outcomes, and long term health for offspring, such as metabolic disease, childhood cancer, schizophrenia, autism spectrum disorder, and other behavioral problems.^[18–21]^

It is known that macrosomia is not only associated with the risk of perinatal problems such as intrauterine death, shoulder dystocia, operative deliveries, and fetal hypoxia, but also long term health outcomes such as adult obesity and cardiovascular disease.^[22–24]^ Risk factors for fetal macrosomia include maternal obesity, diabetes, post-term gestation, and advanced maternal age.^[25]^ Although some previous studies have shown an association between paternal age and LBW,^[26,27]^ few studies investigated the association between paternal age and macrosomia.^[28,29]^ A recent study demonstrated that advanced paternal age increased the risk of LBW among preterm infants and for macrosomia among term infants, which is consistent with the results of this study.^[28]^

There are several limitations to this study. First, this study was not able to access values for maternal BMI, before and after pregnancy, underlying disease, and obstetric complications such as gestational diabetes or pregnancy associated hypertension, and family income level, lifestyle factors including physical activity, smoking, and diet, which can affect term neonatal birth weight.^[30–33]^ However, to avoid the influence of complicated pregnancies, we did not include births at 37 weeks’ gestation because complicated pregnancies are more likely delivered as soon as they reach 37 weeks’ gestation. More qualified national birth registry may be needed to improve not only for the study but also for the maternal-neonatal healthcare policies in the future. Second, this study did not include data about family history of LBW or macrosomia. Third, this study did not include neonatal morbidities and long-term health data for neonates. However, this study demonstrated association between parental age and LBW and macrosomia in term neonates, with data from a National Database of Korea. Notably, this study revealed that not only advanced maternal age, but also advanced paternal age is associated with LBW and macrosomia in term neonates.

This study suggests that both of maternal and paternal age are independent influencing factors for LBW and macrosomia in term neonates. In addition to age, parents’ education level decreased the risk of LBW and macrosomia in term neonates. Therefore, we can hypothesize that the socioeconomic level of parents can affect neonatal birth weight and most likely other neonatal outcomes. Socioeconomic support and policies that can promote pregnancies in young couples need to be more strengthened, together with providing support for pregnancies in less fertile couples.

### Author contributions

Conceptualization: Hyun Sun Ko.

Data curation: Hyun Sun Ko.

Formal analysis: Yoo Hyun Chung, Hyun Sun Ko.

Writing – original draft: Yoo Hyun Chung.

Writing – review and editing: In Sun Hwang, Gyul Jung, Hyun Sun Ko.
